# The HicAB System: Characteristics and Biological Roles of an Underappreciated Toxin-Antitoxin System

**DOI:** 10.3390/ijms252212165

**Published:** 2024-11-13

**Authors:** Josefa Encina-Robles, Valeria Pérez-Villalobos, Paula Bustamante

**Affiliations:** Molecular and Cellular Microbiology Laboratory, Instituto de Ciencias Biomédicas, Facultad de Ciencias de la Salud, Universidad Autónoma de Chile, Santiago 8910060, Chile

**Keywords:** toxin-antitoxin systems, HicAB, HicA, HicB, biofilm, persistence, phage defense, virulence, plasmid maintenance

## Abstract

Small genetic elements known as toxin-antitoxin (TA) systems are abundant in bacterial genomes and involved in stress response, phage inhibition, mobile genetic elements maintenance and biofilm formation. Type II TA systems are the most abundant and diverse, and they are organized as bicistronic operons that code for proteins (toxin and antitoxin) able to interact through a nontoxic complex. However, HicAB is one of the type II TA systems that remains understudied. Here, we review the current knowledge of HicAB systems in different bacteria, their main characteristics and the existing evidence to associate them with some biological roles, are described. The accumulative evidence reviewed here, though modest, underscores that HicAB systems are underexplored TA systems with significant potential for future research.

## 1. Introduction

Toxin-antitoxin (TA) systems are small genetic elements, not essential for normal bacterial growth, which are abundant in prokaryotic genomes [1,2], and commonly associated to mobile genetic elements (MGEs), both chromosomal (e.g., genomic islands) or extrachromosomal (e.g., plasmids) [3]. Typically, they consist of two genes in an operon coding for a stable toxin whose overexpression affects bacterial growth, and a usually unstable antitoxin that avoids toxin toxicity [4]. TA systems are diverse in genetic organization, regulation, toxin neutralization and toxin targets [5]. Hence, based on the nature and mode of action of antitoxins, currently eight TA system types are recognized [6].

In particular, type II TA systems, thought to be the most abundant and diverse in bacterial genomes [7,8], are organized as bicistronic operons that code for proteins capable of interacting through a nontoxic complex [7]. TA operon promoters are usually repressed by the antitoxin or the TA complex [5], and in the operon, the antitoxin gene precedes the toxin gene, so translation of the corresponding bicistronic transcript produces an excess of antitoxin, which is essential for controlling toxin activity. However, exceptions to this genetic organization are observed in some type II TA systems [9,10]. Under stress conditions, the TA system is activated, leading to free toxins being able to reach their cellular targets and to induce toxicity [11,12].

Classification of type II toxins and antitoxins is based on amino acid sequence similarity and three-dimensional structures [7,13,14], encompassing toxin families such as ParE/RelE, CcdB/MazF, GNAT-fold toxins [15] and HicA [16]. Commonly, each toxin family is associated with a specific antitoxin family [7], but hybrid associations have been also characterized [17].

Since their first characterization, multiple biological roles have been associated to TA systems, including some controversial [18]. However, currently there is consensus for their roles in growth diminution during stress, phage inhibition, MGE maintenance and biofilm formation [19].

Here, we review the current knowledge of the HicAB system, a type II TA system that, although ubiquitous in bacterial genomes, has been less studied and little is known about its biological roles.

## 2. Type II HicAB System

**Figure 1:**
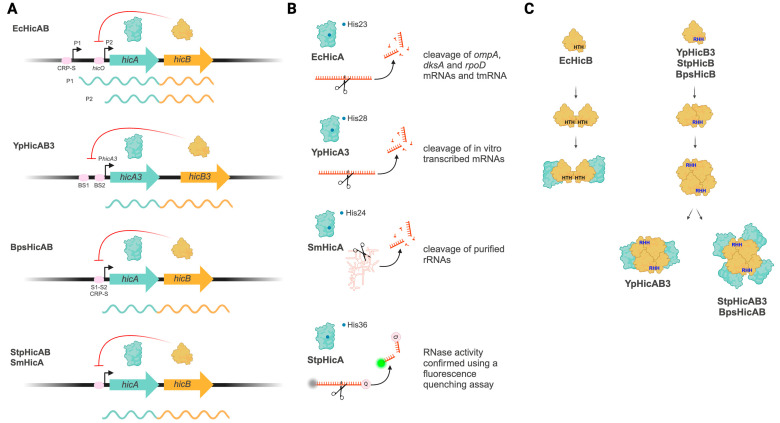
Genetic organization and main characteristics of characterized HicAB systems. Characteristic of *E. coli* HicAB (EcHicAB), *Y. pestis* HicAB3 (YpHicAB3), *B. pseudomallei* HicAB (BpsHicAB), *S. meliloti* HicAB (SmHicAB) and *S. pneumoniae* HicAB (StpHicAB) are included in the scheme. (**A**) Genetic organization of *hicAB* operons. *E. coli hicAB* has two promoters (P1 and P2). P1 contains a CRP-binding site (CRP-S), and it allows expression of both toxin and antitoxin genes. P2 contains the operator *hicO* and it is repressed by EcHicB antitoxin; when it is active, P2 allows expression of the antitoxin only. In *Y. pestis hicAB*, a unique upstream promoter contains two operator sequences (BS1 and BS2) to which YpHicB3 binds. *B. pseudomallei hicAB* possesses a putative upstream promoter with palindromic DNA binding sites (S1–2) for BpsHicB binding; S1—S2 overlap with a predicted CRP-S motif. For *S. pneumonia* and *S. meliloti hicAB* operons, HicB antitoxins were shown to bind their putative promoter, but specific binding sites are unknown. Bicistronic transcripts from Bps*hicAB*, Stp*hicAB* and Sm*hicAB* have not been validated. The HicA effects on HicB DNA-binding are omitted; for details, see the main text. Red curved arrows indicate inhibition. (**B**) HicA toxins and their described RNase activities. The experimental evidence demonstrating RNase activity for each toxin is indicated. Important catalytic residues for each HicA toxin are schematized by blue dots. (**C**) HicB and HicAB complex formation. HicB antitoxins have HTH (EcHicB) or RHH DNA-binding domains. EcHicB forms a single dimeric DNA-binding unit able to interact with two HicA toxins. On the contrary, RHH antitoxins (YpHicB3, StpHicB and BpsHicB) form tetramers (a dimer of dimers), containing two exposes RHH DNA-binding domains, and they interact with HicA toxins forming both heterohexameric (YpHicAB3) or heterooctameric (StpHicB and BpsHicB) complexes. Schemes are not to scale. Created in BioRender.com.

*hicAB* was first described in 1998 as a gene locus inserted into the major pilus gene cluster in several strains of *Haemophilus* [16], and it was not until after approximately a decade that it was identified as a type II TA system, HicA and HicB being the toxin and antitoxin components, respectively [20,21]. Contrasting with canonical type II TA systems, *hicAB* loci are characterized by an inverted genic organization (Figure 1A) [9], with the toxin gene preceding the antitoxin gene in the TA operon.

Pioneering comparative-genomic analysis revealed that the *hicA* and *hicB* genes are abundant in free-living archaea and bacteria, with many genomes containing multiple copies of each [20,21]. Originally, ~230 HicA family proteins and almost twice as many (~450 sequences) HicB proteins were detected in most major clades of bacteria and archaea [20]. Another study reported 119 *hicAB* loci among 218 bacterial and archaeal genomes [21]. However, HicAB systems appear to be more abundant than previously thought, as currently the TADB 3.0 database [8] includes 8,408 TA systems belonging to the HicAB family in different bacteria; of them, just a few have been experimentally validated (Figure 1), including systems from *Escherichia coli* (EcHicAB) [21,22], *Streptococcus mutans* (StmHicAB) [23], *Burkholderia pseudomallei* (BpsHicAB) [24,25], *Yersinia pestis* (YpHicAB3) [26], *Pseudomonas aeruginosa* (PaHicAB) [27], *Streptococcus pneumoniae* (StpHicAB) [28], *Acetobacter pasteurianus* (ApHicAB) [29,30] and *Sinorhizobium meliloti* (SmHicAB) [31]. In addition, the toxicity and neutralization capability of the antitoxin was recently reported for a HicAB system from the cyanobacteria *Anabaena* sp. PCC 7120 [32].

While most of the work about HicAB systems has consisted of functional and structural analysis, only a few papers focused on the study of their biological role, as we review here.

### 2.1. HicA Toxins

Type II TA toxins target a wide variety of cellular processes to interfere with essential functions and inhibit bacterial growth. Some toxins modify their cellular targets post-translationally [33], for example by phosphorylation [34], AMPylation [35] or by acetylation of target tRNAs [36]. However, RNases make up a large proportion of type II toxins that impede protein translation through the cleavage of mRNAs (both by a ribosome-dependent or -independent mechanism), rRNAs or tRNAs, even though their structures, specificities and mechanisms differ [11,37].

HicA proteins (COG1724) correspond to small (50–100 amino acids), mono-domain proteins with a characteristic double-stranded RNA-binding domain (dsRBD fold) [20], and they are nucleases that degrade RNAs in a ribosome-independent manner. The first experimental evidence of HicA as the toxin component of the TA system came from evidence in *E. coli* K-12 [21]. *E. coli hicA* encodes a 58-amino-acid protein whose ectopic production is bacteriostatic, resulting in a rapid cessation of cell growth and simultaneously inhibited cell proliferation [21].

RNase activity has been experimentally demonstrated for EcHicA [21], YpHicA3 [26], SmHicA [31] and StpHicA [28] (Figure 1B). The ectopic production of EcHicA produced a strong inhibition of the rate of translation and it induced mRNA cleavage in three different test mRNAs (*ompA*, *dksA* and *rpoD*) and tmRNA [21]. On the other hand, purified YpHicA3 and SmHicA were able to degrade in vitro-transcribed mRNAs [26] and purified rRNAs [31], respectively. Nevertheless, HicA substrate specificities and consensus recognition motifs, as well as the precise targets of HicA in vivo, are unknown.

Amino acid residues important for HicA activity have been identified by site-directed mutagenesis. For instance, His28 in YpHicA3 is an important catalytic residue involved in RNase activity [26], and Gly22 along with the highly conserved His24 residue (corresponding to Gly26 and His28 in YpHicA3) are required for BpsHicA toxicity, and the authors suggest that they probably have important roles in the HicA–substrate complex [25]. An enzymatic activity has not been demonstrated for BpsHicA, although essentiality of its His24 residue for toxicity suggests an RNase activity [25]. On the other hand, by sequence alignment and structural similarity, a His36 residue in the active site of StpHicA was identified and experimentally validated, as a key residue for catalysis and toxicity [28]. In EcHicA and SmHicA, the histidine residues (His23 and His24, respectively) important for RNase activity are conserved [31].

### 2.2. HicB Antitoxins

Through their DNA binding domains TA antitoxins can bind their TA promoters to repress transcription. Hence, type II antitoxins have two functions: they neutralize their cognate toxins through direct binding and repress toxin gene transcription [38].

HicB antitoxins (COG1598/4226) have a partial RNase H fold in their N-terminus, and either an HTH or RHH DNA-binding domain at their C-terminal end (Figure 1C) [20]. According to sequences and available HicB crystal structures, it has been shown that across species, HicB antitoxins resemble each other in their N-terminal domains (which neutralize HicA), while they differ significantly in their DNA-binding domains and dimerization interfaces [39]. For instance, EcHicB [20,39] has an HTH DNA-binding domain, while YpHicB3 [26], StpHicB [28] and BpsHicB [40] contain an RHH DNA-binding domain [39]. Notably, dimerization of EcHicB occurs using a β-strand following the core HTH motif [39], whereas YpHicB3, StpHicB and BpsHicB all dimerize using the entire RHH fold [26,28,40]. Thus, the dimeric EcHicB has two separate DNA-binding domains, while the RHH HicB antitoxins form a single, dimeric DNA-binding unit (Figure 1C).

Transcriptional repression of the TA operon by HicB antitoxins has been experimentally demonstrated by promoter–reporter fusion [9,26], and direct binding of HicB antitoxins to their TA promoters has been shown for YpHicB3 [26], EcHicB [9,39], SmHicB [31], StpHicB [28] and BpsHicB [40] (Figure 1A).

In *Y. pestis*, sequence analysis of the promoter *hicA3* region revealed the presence of two 15 bp inverted repeats (BS1 and BS2), and HicB3 bound to DNA fragments bearing either BS1 or BS2, but not when both sites were mutated simultaneously [26]. YpHicB3 forms a tetramer (a dimer of dimers), where dimers bind through their N-terminal domains [26]. The YpHicB3 tetramer (as well as the YpHicAB3 complex) bound to 15 bp operators flanking P*hicA3* in vitro and repressed P*hicA3* in vivo [26].

Using a combination of datasets (crystal structures from purified full length HicB, a mutant HicB-I51M/I99M and a HicB N-terminal domain construct), the complete structure of BpsHicB was solved by molecular replacement. Similar to YpHicB3, BpsHicB forms a tetramer with RHH domains exposed for DNA binding and two of four HicA-interacting helices tucked away [40]. In vitro assays revealed that BpsHicB binds a region with a single palindromic sequence that overlap with the predicted −10 sequence and cAMP receptor protein (CRP)-binding sites at the P*hicAB* sequence (Figure 1A).

The crystal structure of the StpHicAB complex revealed that the C-terminal DNA-binding domain of the HicB antitoxin shows considerable structural variation and like BpsHicB it is structured as homodimers able to interact to form tetramers [28] (Figure 1C).

Functional studies revealed that in *E. coli*, *hicAB* expression is controlled by two promoters and one of these is regulated in the canonical way by EcHicB binding and gives rise to a transcript that produces only the antitoxin [9] (Figure 1A). EcHicB binds to DNA with different kinetics and at lower affinity compared with the EcHicAB complex [39]. Indeed, contrary to the EcHicAB–DNA complex, purification of the EcHicB–DNA complex using size-exclusion chromatography was unsuccessful, supporting that HicB forms a relatively unstable protein–DNA complex [39].

Distinct DNA-binding sites are recognized by each characterized HicB protein, suggesting that HicB proteins from different species can recognize different DNA motifs.

### 2.3. HicAB Complexes

**Figure 2:**
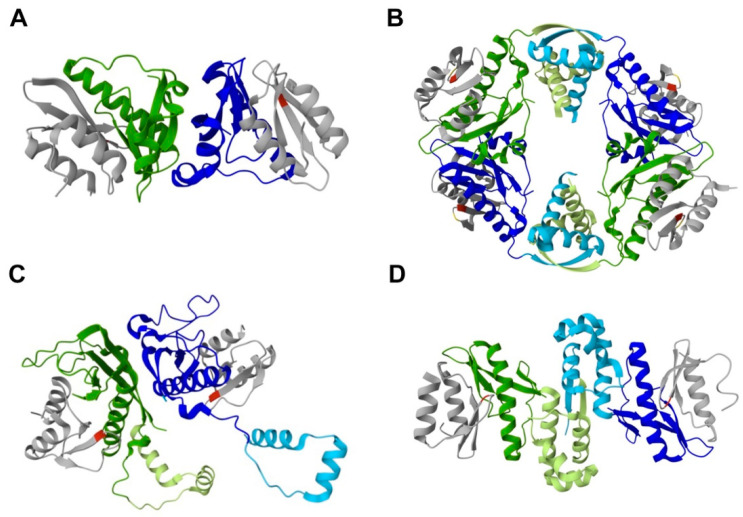
Cartoon representations of HicAB complex crystal structures. (**A**) YpHicAB3 (PDB: 4P78), lacking the C-terminal domain of YpHicB3, forms a heterotetramer, containing two copies of YpHicA3 and YpHicB3. (**B**) BpsHicAB (PDB: 6G26) is a heterooctamer, composed by four HicA and four HicB subunits. (**C**) StpHicAB (PDB: 5YRZ) and (**D**) EcHicAB (PDB: 6HPB) complexes form heterotetrameric assemblies, formed by two copies of both HicA and HicB. HicB subunits are in green and blue; HicA subunits are in grey; important catalytic residues for each HicA toxin are in red (His) or yellow (Gly22 in BpsHicAB); HicB C-terminal DNA-binding domains are highlighted in light green or light blue.

A key characteristic of type II TA systems is the formation of a protein complex, where toxin and antitoxin interact to prevent the toxin from reaching its cellular target and causing toxicity. Bacterial TA complexes have distinct toxin/antitoxin ratios and tridimensional arrangements. Heterohexameric and heterooctameric rearrangements have been observed for HicAB complexes (Figure 1C and Figure 2).

The first X-ray structure reported for a HicAB family complex was for YpHicAB3 [26], followed by the structures of the BpsHicAB [40], StpHicAB [28] and EcHicAB [39] complexes (Figure 2).

The YpHicAB3 complex, lacking the C-terminal domain of YpHicB3, forms a heterotetramer containing two copies of HicA3 and two copies of HicB3 (Figure 2A) [26]. However, the experimental evidence is more compatible with a heterohexameric YpHicAB3, composed of a tetramer of YpHicB3, with two RHH DNA-binding folds able to receive two YpHicA3 molecules (Figure 1C). On the other hand, elucidation of the complex structure of BpsHicAB revealed that it is a heterooctamer, composed of four HicA and four HicB subunits, with four identical HicA interaction sites at the N-domains of HicB (Figure 2B) [40]. Similarly, the StpHicAB complex model corresponds to a heterooctamer that contains dimeric interfaces between the HicB chains (Figure 1C). However, the StpHicAB complex crystal structure contains two HicB antitoxins and two HicA toxins which form a heterotetrameric assembly (Figure 2C); Phe22 or Glu47 were identified as key residues essential for the interaction between StpHicB and StpHicA [28]. EcHicAB’s structure differs significantly from other structures of HicAB complexes that contain an RHH-type DNA-binding domain; the EcHicAB complex is formed by two copies of both HicA and HicB, forming a heterotetrameric complex [39]. This tetramer forms by interaction of two nearly identical HicA–HicB complexes, through dimerization of the C-terminal domains of HicB (Figure 2D) [39].

In HicAB complexes, the His residues required for HicA RNase activity and toxicity, for instance His28 in YpHicA3 [26] and His24 in BpsHicA [40], are situated completely masked at the interface with HicB. Thus, HicB antitoxins, regardless of their DNA-binding domain, neutralize HicA toxins by blocking their active site.

As we mention before, free HicB antitoxins or HicAB complexes can bind DNA to repress their TA promoters (Figure 1A). The YpHicAB3 complex can bind to a 365 bp DNA fragment bearing P*hicA3* in vitro and repress P*hicA3* in vivo [26]. The StpHicAB heterooctamer interacts with dsDNA and the HicAB complex has a higher affinity for DNA than HicB [28].

## 3. Regulation of *hicAB* Loci Expression

One of the established biological roles of TA systems is growth diminution during stress, and in consequence several stress conditions are known to activate TA systems. For instance, transcription of *hicAB* in *E. coli* is induced by amino acids and carbon starvation by a Lon-dependent mechanism, and by chloramphenicol [21]. On the other hand, *hicA* from *B. pseudomallei* was not upregulated when the bacteria were within the macrophage [41]. However, transcription of TA operons not necessary imply toxin activation [42], and it has been argued that toxins bound to antitoxins are not likely to become activated by preferential antitoxin degradation but instead, de novo toxin synthesis in the absence of stoichiometric amounts of antitoxin activates toxins [43]. Thus, validation of HicA toxin activation is necessary under the experimental stress conditions analyzed.

In canonical type II TA systems, the TA operon adopts a genetic organization with the antitoxin gene as the first gene in the operon and regulation relies on a conditional cooperativity mechanism [44,45]. Interaction with DNA relies on antitoxins binding to operator sequences and toxins can be either a corepressor or a derepressor, depending on the toxin/antitoxin ratio; when the toxin level exceeds the antitoxin level, repression is alleviated. However, there are exceptions with an inverse genetic organization [2] or without conditional cooperativity [46,47]. For systems with an inverse genetic order, toxins were shown to displace their cognate antitoxins from operator sequences upon binding and not act as corepressor.

Canonical *hicAB* loci have an inverse genetic organization, like *mqsRA*, *rnlAB* and *higBA* systems [2]. For those, a common mechanism of regulation by alternative promoters was suggested, to avoid harmful excess of toxin. An upstream promoter allows for expression of both toxin and antitoxin genes, while downstream promoters exclusively drive the expression of the antitoxin gene [9,10,48]. However, for HicAB systems a double promoter mechanism has been shown only for EcHicAB [9], and a recent bioinformatic analysis [49] revealed that HicA domains could be found in at least 14 distinct genetic contexts, including *hicAB* and *hicBA* operons, suggesting that different regulatory mechanisms could control *hicA* expression. Indeed, the presence of HicB proteins that lack a DNA-binding domain is common [49].

In *Y. pestis*, a 174 bp intergenic region separates *hicA3* and *hicB3* in the bicistronic operon; a promoter is present upstream of *hicA3* (P*hicA3*) and no constitutive promoter was detected in the intergenic region [26]. Reporter fusion experiments and gel shift assays indicated that the YpHicB3 antitoxin, as well as the YpHicAB3 complex, repress and bind to two operators (BS1 and BS2) in the P*hicA3* region [26]. However, in vivo data showed that although YpHicB3 can bind both the BS1 and BS2 sites, the main operator of P*hicA3* is BS2, which overlaps with the −10 box [26]. However, whether a conditional cooperativity mechanism controls P*hicA3* expression remains elusive, although it was suggested that an excess of YpHicA3 destabilizes the YpHicAB3–DNA complex and titrates out the YpHicB3 repressor [26]. Similarly, in the MqsRA system, the toxin MqsR is not a corepressor, but destabilizes the MqsA–DNA repression complex via allosteric modification [46].

In *B. pseudomallei*, binding of BpsHicA to BpsHicB induces a structural reorganization that disrupts the DNA binding sites to expose the BpsHicA-interacting helices [40], giving a rationale of how HicA can derepress HicB–operator binding at high toxin/antitoxin ratios [40].

In contrast, no major structural rearrangements are required for the formation of the HicAB complex in *E. coli* [39]. EcHicB was shown to interact weakly with a 147 bp dsDNA fragment containing the upstream region of *hicA*, and this interaction was sufficient to cause repression, as the toxin HicA did not affect this binding significantly; however, an excess of HicA derepresses the EcHicB–DNA complex and restores transcription of EcHicB [9].

Among the characterized HicAB systems, the double promoter mechanism has been studied in detail only for EcHicAB [9]. The *E. coli hicAB* locus has two promoters (Figure 1A); the upstream promoter (P1) allows for expression of both toxin and antitoxin genes and the downstream promoter (P2) produces an mRNA that only allows for expression of the antitoxin [39]. Both promoters are regulated by different mechanisms; the upstream promoter contains a CRP-S motif and is activated by Sxy, while the downstream promoter is repressed by EcHicB, and this repression is relieved upon excess of EcHicA [39]. This mechanism allows for the specific production of antitoxin when the toxin/antitoxin ratio becomes too high and thus prevents activation of the system under normal growth conditions. As was mentioned before, EcHicB does not undergo any major structural rearrangements upon binding of EcHicA [39], which is consistent with the observation that the toxin does not function as a corepressor of HicB in vivo [9], similar to YpHicA [26].

## 4. Biological Role of HicAB Systems

The genetic and structural diversity among TA systems is also extrapolated to their biological roles. They have been associated to several biological processes, even some controversial [18]. One of the established biological roles of TA systems is growth diminution during stress, in addition to phage inhibition, MGE maintenance and biofilm formation [19].

Among HicAB systems, there are only a few studies reporting a biological role or their involvement in a physiological process, showing that HicAB could be associated with bacterial persistence, biofilm formation, virulence and a proposed role in anti-phage defense (Table 1) [22,25,26,30,49,50]. In addition, a HicAB system has been used as a counterselection marker for the development of a molecular biology tool [51].

### 4.1. Biofilm Formation

Biofilms are aggregates of microorganisms in which cells are embedded in a self-produced matrix of extracellular polymeric substances (EPSs) that are adherent to each other and/or a surface, and they provide protection against antibiotics and evasion of host innate immunity [52,53].

Several type II TA systems have been reported to be involve in biofilm formation, although there is contrasting evidence. For example, the deletion of the *mazEF* system reduced biofilm formation in *E. coli* [54], contrasting with the increased biofilm formation of a *mazF* deletion mutant in *Staphylococcus aureus* [55]. Similarly, the deletion of the *relBE* system reduced biofilm formation in *E. coli* [56], *Vibrio cholerae* [57] and *S. pneumoniae* [58]; however, *relE* deletion had no effect on biofilm formation in *Streptococcus mutants* [59]. On the other hand, the antitoxin MqsA was reported as a global regulator that reduces expression of several stress response genes, comprising the repression of curli formation through the master biofilm regulator CsgD [60]. However, other authors have reported that the MqsRA system does not contribute to the regulation of biofilm formation [10].

A role in biofilm formation for a HicAB system was studied in the extraintestinal pathogenic *E. coli* (ExPEC) PPECC42 strain [22]. In this strain, biofilm formation was significantly reduced in a *hicAB* deletion mutant compared to wild-type strain, but the effect was not related to curli production, or the irreversible adhesion stage [22]. However, the expression of outer membrane proteins and genes involved in cellulose synthesis were upregulated in the *hicAB* mutant [22]. These results suggested that the HicAB system seems to be essential for biofilm formation in ExPEC, and the effect might not be associated with reduced extracellular matrix production, but with changes in the expression of outer membrane proteins and cellulose synthesis, which is known to be related to biofilm formation in *E. coli* [61].

In *B. pseudomallei*, deletion of *hicA* resulted in significant reduction in biofilm formation [41]. In *B. pseudomallei*, the formation of biofilm is an indicator of the propensity of bacteria to generate persister cells, allowing for multidrug tolerance.

On the other hand, deletion of *hicAB* in *P. aeruginosa* had no effect on the biofilm formation [27].

### 4.2. Bacterial Persistence

Persister cells represent a subpopulation of slow-growing bacterial cells with decreased susceptibility to being killed by bactericidal antibiotics, while they are not antibiotic-resistant [62]. Molecular mechanisms of persister cell formation are elusive; it is noteworthy that TA systems have long been linked to bacterial persistence, although this biological role is controversial [63,64,65].

Until now, the role of HicAB systems in persister cell formation has been studied for BpsHicAB and ApHicAB.

The effect of BpsHicA on *E. coli* persister cell formation was assayed after *hicA* overexpression, and the bacteria increased the frequency of surviving ciprofloxacin and ceftazidime exposures [25]. However, *hicAB* deletion reduced the number of ciprofloxacin persisters, but did not affect the numbers of bacteria surviving a ceftazidime treatment [25]. Residues important for BpsHicA toxicity, His24 and Gly22 (see Section 2.1), were also important for the persistence phenotype [25]. Another study shows no significant differences in the survival of a *B. pseudomallei hicA* deletion mutant, compared with the wild type, following the exposure to ceftazidime or levofloxacin [41]. However, the *hicA* deletion mutant had reduced rates of persister cells following macrophage internalization [41].

A recent report showed in *A. pasteurianus*, an acetic acid bacteria, that the HicAB system contributes to the acetic acid production, persister formation and survival of bacteria in a highly acidic environment [30].

### 4.3. Virulence

Since TA systems can be involved in various physiological processes, it is important to note that they may influence virulence of bacterial pathogens. In relation to HicAB systems, a role in virulence has been studied for YpHicAB, BpsHicAB and PaHicAB.

In a murine model of bubonic plague, *Y. pestis hicB3* was upregulated relative to in vitro bacterial cultures [66], and a *hicB3* deletion mutant had attenuated virulence [50]. However, in a later report, a Δ*hicAB3* mutant was fully virulent in the same murine model [26]. So, the authors concluded that previous results with a Δ*hicB3* mutant were probably due to inefficient in vivo growth caused by the activity of the free HicA3 toxin, and a role of YpHicAB in virulence is unlikely.

Similarly, a role in virulence for the BpsHicAB and PaHicAB systems was discarded. In *B. pseudomallei*, a *hicA* deletion led to reduced ability to colonize the lung in a murine model of melioidosis, but the spleen was colonized to the same extent as wild-type bacteria; however, the *hicA* deletion mutant led to mouse mortality comparable to the wild-type strain [41]. In *P. aeruginosa*, the survival rate of BALB/c mice infected with a *hicAB* deletion mutant was comparable to the survival of mice infected with the wild-type strain [27].

The experimental evidence available until now, in different bacteria, allows to discard a role in virulence for the HicAB system.

### 4.4. Phage Defense

Currently, a renowned biological role of TA systems is to act as defense systems that inhibit the spread of phages, by an abortive mechanism [67,68]. There are several type II TA systems whose role in phage inhibition has been demonstrated, including MazEF that inhibits phage P1 [69] and RnlAB that inhibits T4 phage [70]. A recent publication proposed a role in anti-phage defense for HicAB systems, based on genetic contexts of HicA toxin domains [49].

The *hicA* genes are occasionally closely associated with genes coding prokaryotic viperin proteins (pVIPs) [49], orthologs of eukaryotic proteins that have anti-phage activity [71]. Type II *relE* toxin genes were also found in a similar context [49]. The author proposed that both HicA and RelE RNases work together with pVIPs to mount a strong defense against phage infections.

Clearly, experimental validation under physiological conditions is necessary to confirm the role of HicAB systems in anti-phage defense.

## 5. HicAB Systems in MGEs

Since its first characterization, the distribution and abundance of the hicAB locus in bacterial genomes suggest that it is transferred by horizontal gene transfer (HGT) [20]. Indeed, several *hicAB* loci are found encoded in prophages and plasmids [20,72,73,74]. In *P. aeruginosa*, it was found that the flanking regions of the chromosomal *hicAB* locus encode several proteins homologous to phage-related proteins, supporting an acquisition by HGT [27].

Currently, seven HicAB systems are reported by the TADB 3.0 database [8] encoded in MGEs, both in prophages, genomic islands and plasmids (Table 2); however, not all of them have been completely validated.

### 5.1. Plasmidial HicAB Systems

The first recognized biological role of TA systems was the maintenance of plasmids as postsegregational killing (PSK) systems, to prevent the accumulation of plasmid-free cells in the population [79]. The first live evidence of the TA-mediated PSK phenomenon was recently shown [80].

Unlike chromosomally encoded TA systems, which have been the major focus of research, plasmid-based TA systems, beyond their identification, are relatively understudied, and studies have mainly focused on antibiotic-resistant plasmids [73,81,82].

An analysis of the distribution of TA systems on bacterial genomes exposed that *hicAB* loci are rarely encoded by bacterial plasmids [83]. Surprisingly, *hicAB* loci are highly common on a particular subfamily of plasmids, the IncX4 group, which most confer antibiotic resistance to carbapenems and colistin [73,84,85], the last-resort antibiotics for multidrug-resistant Gram-negative pathogens [86].

To our knowledge, none of the known plasmidial HicAB systems has been characterized. It is noteworthy that a plasmid maintenance role was discarded for one of them [78].

The association of HicAB systems with a specific family of plasmids suggest specialization, and these TA systems might be undertaking specific roles on their plasmids. These roles might be alternative to plasmid maintenance, as has been demonstrated for other plasmidial TA systems [87].

### 5.2. HicAB Systems in Bacteriophages

As was stated before (see Section 4.4), phage defense is a bona fide function of TA systems [68]. It is noteworthy that phages have evolved sophisticated mechanisms to counteract TA systems, and among them, the acquisition of antitoxins or antitoxin mimics is usual [68]. Consequently, the identification of TA-like systems is not uncommon in bacteriophage genomes [24,27,72,75,77], although their roles are poorly defined.

A HicAB system was identified in a *Paracoccus* spp. prophage, and its functionality as a plasmid stabilizing system was proven [75]. On the other hand, the BpsHicAB system involved in persister cell formation [25] is encoded in a bacteriophage as part of a genomic island [24].

Recently, a HicAB-like TA system from the *Mycobacterium* phage Adephagia was identified and characterized [74]. Adephagia is a temperate phage that infects *Mycobacterium smegmatis* and some strains of *Mycobacterium* pathogens [88]. Transcriptomic analysis of an Adephagia lysogen revealed a high expression of a gene coding for a HicB-like antitoxin (gp90), and low expression levels of a gene which codes for a putative HicA-like toxin (gp91); additionally, expression of the gp91 toxin is highly toxic to *M. smegmatis*, and a Δ*90* mutant was defective in lytic growth and plaque formation [74]. Protein alignments and structure prediction of Adephagia gp90 and gp91 showed strong similarities to other HicAB proteins, and conservation of a HicA catalytically important histidine residue, His31 [74]. On the other hand, and in agreement with the double promoter mechanism for *hicAB* operon regulation [9], two putative promoters were identified for gene *90* (P_90_) and *91* (P_91_), which might differentially express both genes [74]. Those data highly support Adephagia gp90 and gp91 proteins as the antitoxin and toxin components of a cognate HicAB system, respectively.

The authors proposed that the Adephagia HicAB-like TA system could play a role in prophage maintenance by killing off those phage-cured cells, and alternatively it could confer defense against further phage infection by an abortive mechanism [74]. However, they could not identify any targeting phages among a diverse panel of tested phages.

As it is common to find uncharacterized TA-like systems in bacteriophage genomes, they represent intriguing systems to be studied and to reveal unknown roles of this TA family.

## 6. Conclusions

Although they are abundant in bacterial genomes, HicAB systems remain poorly studied, and their biological roles are still elusive.

Here, we have shown that this TA family shows non-cognate characteristics, and they represent an attractive source to uncover unknown biological roles. For instance, there is evidence for a role of HicAB in biofilm formation and persistence, but phage defense and antibiotic-resistant plasmid maintenance are also feasible. On the other hand, a role in virulence was discarded based on experimental evidence, although we cannot rule out this role for all HicAB systems, essentially because only a couple of systems have been characterized. Furthermore, to reveal HicA in vivo RNA targets, undoubtedly will help to decipher which cellular processes are affected by this RNases.

The specific association of *hicAB* loci with a particular plasmid family, in addition to the great diversity and abundance found around *hicA* loci in bacterial genomes, highlight that HicAB (or HicAB-like) systems represent an underappreciated TA system, and attractive for future research.

## Figures and Tables

**Table 1:** Investigated HicAB roles in bacterial cell physiology.

Organism	Biological Role	Reference
ExPEC	Biofilm formation: the ability of a ΔhicAB deletion mutant was significantly decreased, independent of curli formation.	[22]
*B. pseudomallei*	Biofilm formation: *hicA* deletion reduced biofilm formation. Bacterial persistence: *hicA* overexpression increased the fraction of persister cell population that is tolerant to ciprofloxacin and ceftazidime. The deletion of *hicAB* in *B. pseudomallei* reduced the number of ciprofloxacin persisters, but not ceftazidime persisters.	[25,41]
*Yersinia pestis*	Virulence: *Y. pestis* lacking *hicB3* is attenuated for virulence; however, a Δ*hicAB3* mutant is fully virulent, discarding a role of YpHicAB in virulence.	[26,50]
*Pseudomonas aeruginosa*	Biofilm formation: deletion of *hicAB* had no effect on the biofilm formation. Virulence: *hicAB* deletion had no effect on virulence in a mice infection model.	[27]
Several bacteria	Phage defense: bioinformatic analysis identified that *hicA* genes are occasionally closely associated with genes that could have anti-phage activity and it was proposed that they could work together against phage infections.	[49]
*Acetobacter pasteurianus*	Bacterial persistence: HicAB regulates the formation of persister cells responsible for the acid stress resistance.	[30]

**Table 2:** *hicAB* loci found on MGEs and with experimental evidence, according to TADB 3.0.

Organism	Accession Number	TA Loci	MGE	Reference
*Paracoccus phage* vB_PbeS_Pben1	MK291441	pben1_p26 (hicA)/pben1_p25 (hicB)	Prophage	[75]
*Thermotoga maritima* MSB8	NC_000853	TM_RS06645 (hicA)/TM_RS06650 (hicB)	Genomic island	[76]
*Pseudomonas aeruginosa* PA1	NC_022808	PA1S_RS06915 (hicA)/PA1S_RS31585 (hicB)	Prophage	[27]
*Acinetobacter baumannii* ATCC 17978	NC_009085	A1S_2020 (hicA)/A1S_2019 (hicB)	Prophage	[77]
*Burkholderia pseudomallei* K96243	NC_006351	BPS_RS20815 (hicA)/BPS_RS20820 (hicB)	Prophage-IS/Tn	[24,25,41]
*Streptococcus pneumoniae* TIGR4	NC_003028	SP_RS08870 (hicA)/SP_RS08865 (hicB)	IS/Tn	[28]
*Escherichia coli* plasmid pJIE143	JN194214	hicA/hicB	Plasmid	[78]
